# Microarray Noninvasive Neuronal Seizure Recordings from Intact Larval Zebrafish

**DOI:** 10.1371/journal.pone.0156498

**Published:** 2016-06-09

**Authors:** Michaela Meyer, Sameer C. Dhamne, Christopher M. LaCoursiere, Dimira Tambunan, Annapurna Poduri, Alexander Rotenberg

**Affiliations:** 1 Division of Epilepsy and Clinical Neurophysiology, Department of Neurology, Boston Children’s Hospital, Harvard Medical School, Boston, Massachusetts, United States of America; 2 F.M. Kirby Neurobiology Center, Department of Neurology, Boston Children’s Hospital, Harvard Medical School, Boston, Massachusetts, United States of America; 3 Epilepsy Genetics Program, Neurogenetics Program, Department of Neurology, Boston Children’s Hospital, Harvard Medical School, Boston, Massachusetts, United States of America; 4 Neuromodulation Program, Department of Neurology, Boston Children’s Hospital, Harvard Medical School, Boston, Massachusetts, United States of America; Institut Curie, FRANCE

## Abstract

Zebrafish epilepsy models are emerging tools in experimental epilepsy. Zebrafish larvae, in particular, are advantageous because they can be easily genetically altered and used for developmental and drug studies since agents applied to the bath penetrate the organism easily. Methods for electrophysiological recordings in zebrafish are new and evolving. We present a novel multi-electrode array method to non-invasively record electrical activity from up to 61 locations of an intact larval zebrafish head. This method enables transcranial noninvasive recording of extracellular field potentials (which include multi-unit activity and EEG) to identify epileptic seizures. To record from the brains of zebrafish larvae, the dorsum of the head of an intact larva was secured onto a multi-electrode array. We recorded from individual electrodes for at least three hours and quantified neuronal firing frequency, spike patterns (continuous or bursting), and synchrony of neuronal firing. Following 15 mM potassium chloride- or pentylenetetrazole-infusion into the bath, spike and burst rate increased significantly. Additionally, synchrony of neuronal firing across channels, a hallmark of epileptic seizures, also increased. Notably, the fish survived the experiment. This non-invasive method complements present invasive zebrafish neurophysiological techniques: it affords the advantages of high spatial and temporal resolution, a capacity to measure multiregional activity and neuronal synchrony in seizures, and fish survival for future experiments, such as studies of epileptogenesis and development.

## Introduction

Zebrafish have recently emerged as a useful experimental epilepsy model [1–6]. Zebrafish larvae are easy to breed in large quantities, have a well-developed vertebrate central nervous system, share a basic homologue brain organization with all vertebrates, and have high genetic and physiological similarity with humans [7]. Specifically, zebrafish larvae epilepsy models offer several advantages that include rapid development (fish have a fully formed brain and spinal cord 5–7 days post fertilization [dpf]), transparency to visible light, and the easy uptake of compounds added to their surrounding [8, 9]. Thus, they are an efficient model system for developmental studies and for pharmacological screening experiments [10, 11]. Genetic alterations are faster and easier in zebrafish than in other vertebrates and can be made either by creating transgenic lines via mutagenesis (e.g., by N-ethyl-N-nitrosourea, ENU), antisense morpholino oligonucleotides injection, or by application of the clustered regularly interspaced short palindromic repeats (CRISPR)/ CRISPR-associated (Cas) 9 technology [12–17].

Recent studies demonstrate that behavioral assays and electrophysiological field potential recordings in zebrafish can detect seizures [1, 6, 10, 11, 18]. Current methods for *in vivo* zebrafish brain electrophysiological recording involve the use of a single glass electrode positioned either into the intracranial space or on top of the cranium of a paralyzed fish, restrained in agarose [1, 6]. To complement this method, we developed a novel noninvasive technique for transcranial field potential recording with the use of a 61-channel microelectrode array (MEA) and without the embedding of the fish in agarose to improve survival.

To test whether epileptic seizures can be detected by our method, we applied two common chemoconvulsants, potassium chloride (KCl) and pentylenetetrazole (PTZ), to the bath fluid of intact larvae (ages 4–5 days post fertilization, dpf) and compared the recordings with those from untreated wild-type larvae. The use of high extracellular KCl serves as a non-synaptic seizure model where an increase in the K^+^-driving force into the cell leads to membrane depolarization, activation of voltage-gated sodium channels, and subsequent firing of action potentials. PTZ, on the other hand, acts trans-synaptically by blocking gamma aminobutyric acid (GABA)-A receptors and thereby inhibiting dominant inhibitory networks [19].

With our technique, the fish remains viable for repeated recordings and the multiple electrodes enable not only more efficient detection of epileptiform discharges, but also measures of spatial distribution of the recorded signals. MEA systems were initially developed for isolated brain slice and cultured neuron recordings, and are now commonly available cost-efficient bench-top electrophysiological recording systems. Thus, the described method expands the capacity of the neuroscience community to study seizures in zebrafish.

## Material and Methods

### Animals and maintenance

Twenty-two wild-type zebrafish larvae (AB strain) were raised at 28.5°C under standard aquaculture conditions. All animal care procedures were reviewed and approved by the Institutional Animal Care and Use Committee of Boston Children’s Hospital. Animals were monitored daily. Larvae chosen for experimentation did not become ill and did not die prior to the experimental endpoint. Animals did not receive medical treatment but larvae of the same dish as the ones used for experimentation, were humanely euthanized if they exhibited symptoms indicative of illness/moribundity. After experimentation, larvae were first anesthetized with ice cooled fish water and then frozen for euthanasia.

### Electrophysiology

**Fig 1:**
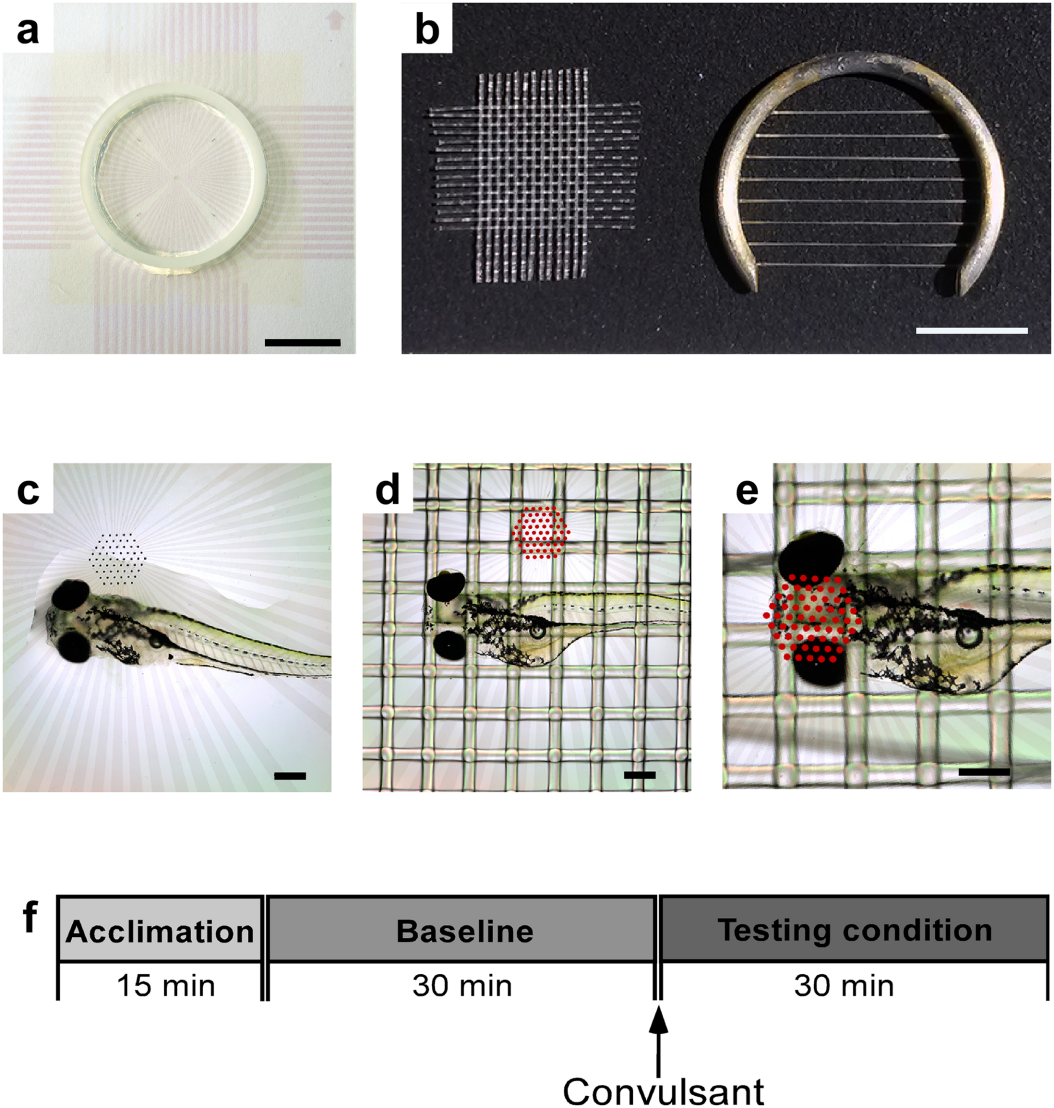
Tools and steps for larva-mounting and timeline of recording. **a:** Electrode chamber used for recordings (scale: 10 mm). **b:** Nylon mesh and slice anchor used to hold larva in place (scale: 5 mm). **c:** Placement of larva onto its dorsal side into the chamber with a drop of water (scale: 300 μm). **d:** Securing larva with nylon mesh (scale: 300 μm); microelectrode array is highlighted with red dots for enhanced visibility in this photo and the next. **e:** Larva positioned with head onto the microelectrode array (scale 300 μm). **f:** Timeline for experiments—each starting with a 15 min acclimation period, a 30 min control recording, and a 30 min recording time after drug application.

Prior to electrophysiological recordings, each larva was anesthetized with ice cold fish water and then transferred to a slice chamber (MED-P2H075; Alpha MED Sciences, Osaka, Japan) of a MED64 System (Fig 1a). Electrodes were 20 μm in diameter and located 70 μm apart from each other; impedance per electrode was 10 kΩ. Fig 1b shows a nylon mesh and a slice anchor for securing the larva.

Each larva was first positioned onto its dorsal side into the chamber (Fig 1c), then secured with the nylon mesh (Fig 1d) and the slice anchor. Subsequently, the head of the larva was positioned onto the electrode grid (Fig 1e). The recording chamber was then carefully filled with carbogenated artificial cerebrospinal fluid (aCSF). The choice to record in aCSF was made due to the delicate structure of the larval cranium and concern for possible penetrance of free water into the intracranial space when the fish head was pressed against the MEA by the slice anchor. The aCSF contained 124 mM NaCl, 3 mM KCl, 26 mM NaHCO_3_, 2 mM CaCl_2_, 1 mM MgSO_4_, 1.25 mM NaH_2_PO_4_, 10 mM Glucose (pH 7.4). The electrode chamber was connected to the MED64 hardware and a constant flow of aCSF to the chamber was established at a rate of 2 ml/min.

The larva acclimated for at least ten minutes at room temperature (RT) before recordings began (Fig 1f). Baseline spontaneous recordings were made for a minimum of 30 min followed by drug addition. We added either 15 mM KCl or 15 mM PTZ to the aCSF (pH 7.4 after addition of drugs maintained by bicarbonate buffer) and recorded for a minimum of 30 min (Fig 1f).

Spontaneous voltage recordings were amplified, band-pass filtered between 0.1 and 100,000 Hz, and digitized using the MED64 hardware at 20 kHz sampling rate per channel.

### Data analysis

We recorded a wide-band local field potential signal, which can contain both extracellular multi-cell action potentials (synonymous terms used in the manuscripts are “spikes” or “units”) and EEG [5]. To isolate multi-unit action potentials, traces were high-pass filtered at or above 100 Hz and spike-number was obtained using window discrimination. To obtain EEG waveforms, raw data traces were band-pass filtered at 1–70 Hz.

Spike times, as well as spike rate, obtained for 1800 seconds for each channel during baseline and after drug application, were extracted from the data set. For quality control, we only analyzed data from experiments that had a minimum of 15 channels showing spontaneous action potentials (defined as “active channels”). Channels that did not show any activity were excluded from analysis. Bursts were defined as spike rates (number of spikes within one second bin) that exceeded twice the standard deviation of the average spike rate and contained a minimum spike frequency of 4 spikes/sec. Averages per fish were computed from spike rate and burst count across all active channels.

While each fish served as an internal control, to test stability and continuity of baseline firing, we additionally completed seven experiments, each 3600 seconds long, of spontaneous recording without adding any drug and analyzed these data the same way as we analyzed individual baseline and drug pairs (as described above).

To assess stability during recordings, twenty to thirty waveforms (3 milliseconds long), each representing a single spike, were averaged. The width and height of the averaged action potentials were taken and compared between baseline and drug conditions.

Based on the list of spike rates per time point per channel, we calculated additional metrics that highlight the spatio-temporal synchrony in the firing patterns. Custom algorithms developed in Matlab (v. 8.2, MathWorks, Natick, MA) were used to determine active firing fraction and spatial coherence. Active firing fraction was defined as the ratio of number of channels with spike rate > 0 to the number of total active channels, per time point. Recordings with a mean percent firing fraction of more than 20% at baseline were considered contaminated by noise and discarded from analysis. Spatial coherence of activity across channels was computed by calculating the r-value equating to the correlation coefficient between each channel’s spike rate (provided the rate was > 0) and the immediately adjacent electrode with the closest-matching spike rate, per time point. Lastly, area under the curve (AUC) of both, firing fraction, and spatial coherence r-values along the time axis, was calculated using numerical integration, and compared between baseline and drug conditions.

### Pharmacological verification of neuronal origin

To verify pharmacologically that recorded waveforms classified as spikes, are indeed brain action potentials and not myogenic potentials, we added 60 μM α-bungarotoxin (Biotium inc. Hayward, CA) to the bath and waited until the animal was paralyzed as determined by absence of spontaneous or mechanically-provoked movement. In parallel with confirming paralysis, we monitored for continuous heartbeat as indication of survival. Then, we obtained recordings at baseline and after 15 mM PTZ addition. After recordings, we checked for survival and paralysis of the animal again. In additional experiments, we added 0.4 mM tetrodotoxin after a minimum of thirty minutes of seizure-recordings in PTZ (Abcam Biochemicals, Cambridge, MA) to the bath.

### Statistics

Statistical analyses were performed using GraphPad Prism (v 6; GraphPad Software Inc,. La Jolla, CA). Paired t-test was used to compare all outcomes of the log transformed spike rate, the log transformed burst count, area under the curve (AUC) for active firing fraction, and spatial coherence between the control and drug conditions.

## Results

### Action potential and EEG recordings

**Fig 2:**
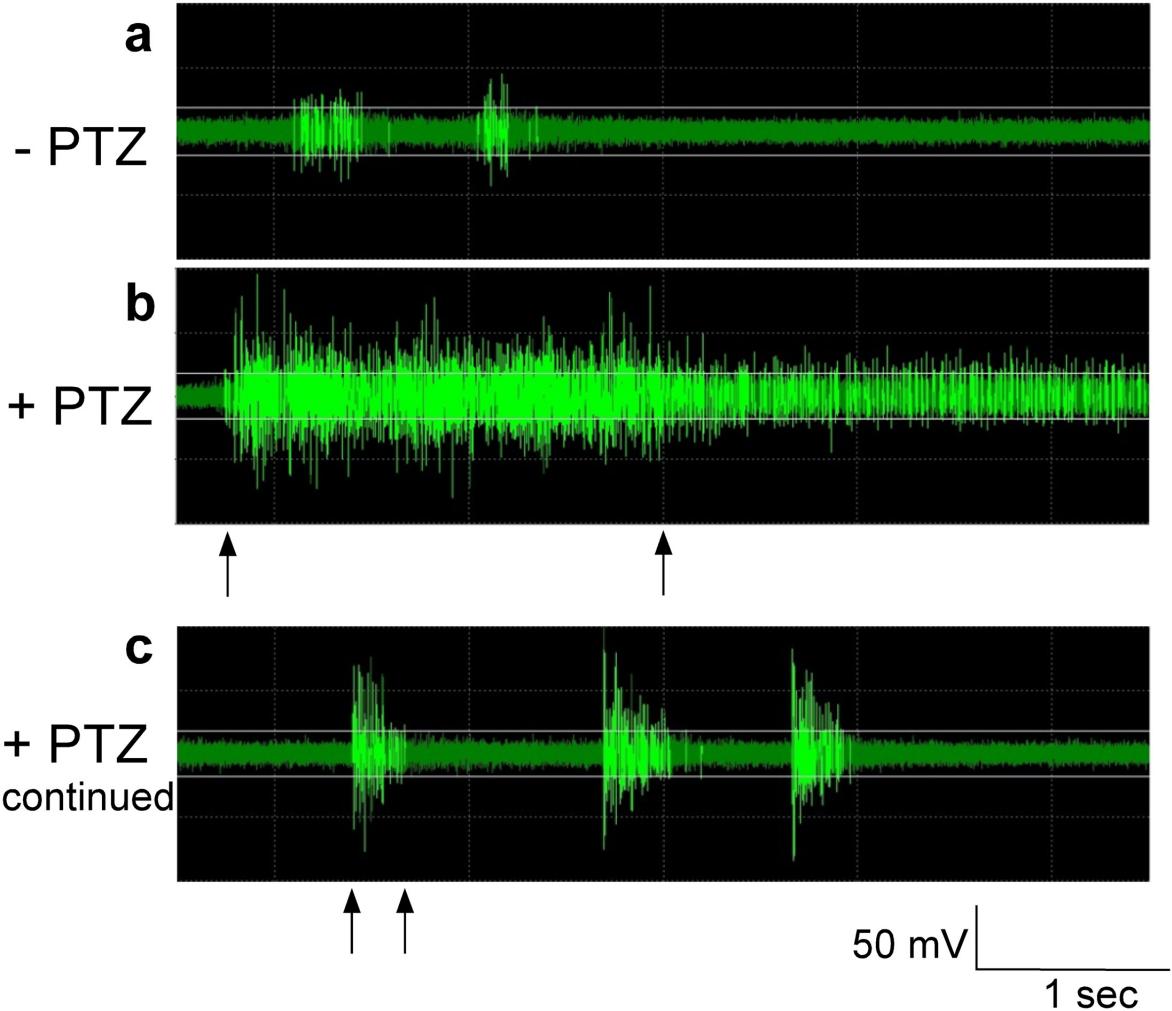
Provoked seizures in 5-second raw data traces of action potentials. **a:** Baseline activity before PTZ addition. **b:** A typical seizure starting with a prolonged action potential burst 1–2 minutes after PTZ application. **c:** Seizures continue as short paroxysmal action potential bursts. PTZ addition leads to a seizure firing pattern. Markings denote sustained (> 2 sec; b) versus short (< 500 ms; c) bursting.

**Fig 3:**
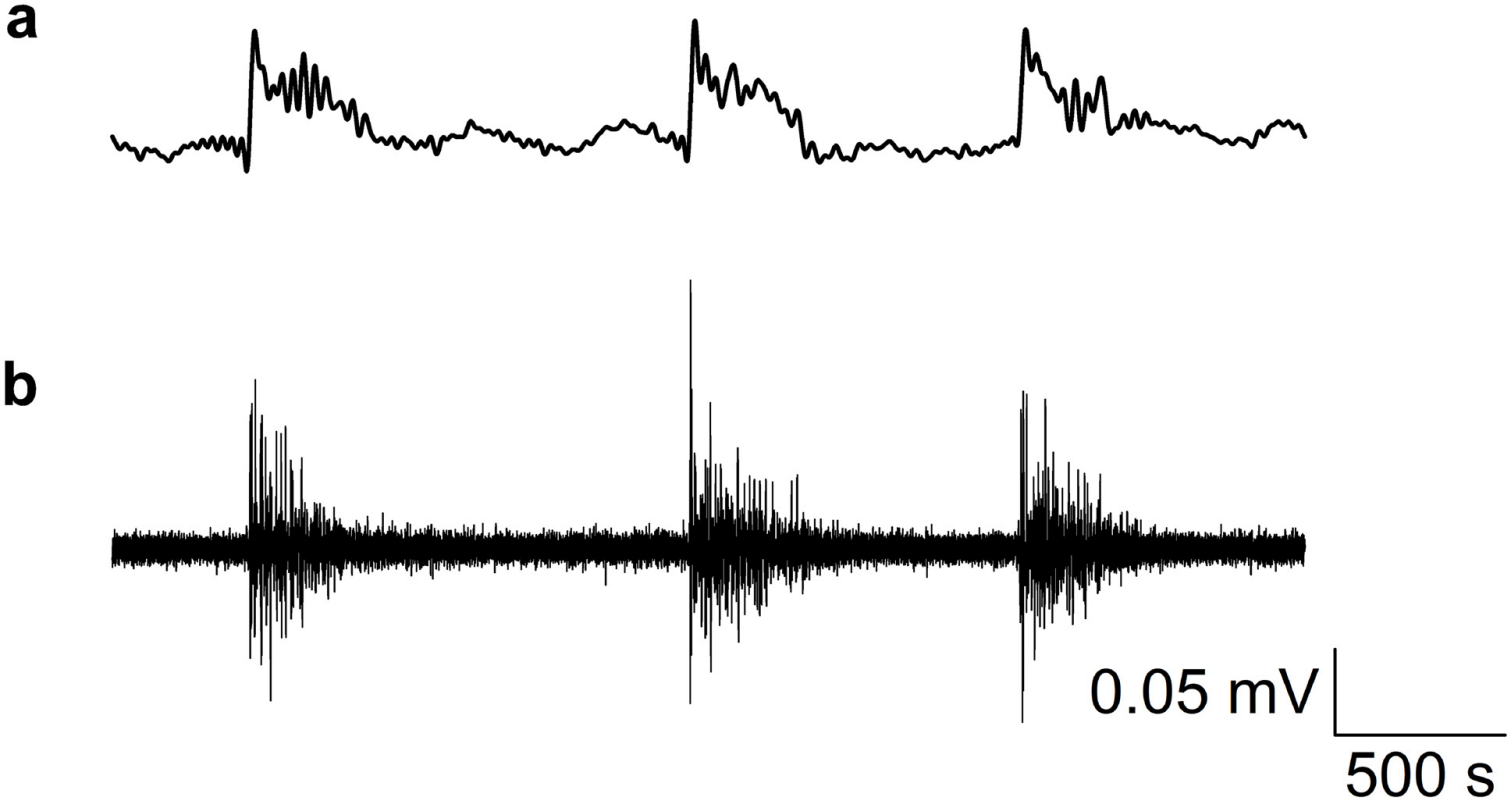
EEG and action potential recordings. Both EEG- and action potential recordings from zebrafish larvae can be obtained simultaneously and correlated with each other. These recordings are taken from one channel following PTZ- administration. **a:** Data are filtered to show an EEG (bandpass at 1 to 25 Hz). **b:** Corresponding multi-unit activity (high pass at 100 Hz).

We recorded from a total of 22 larvae. Multi-unit extracellular action potentials were reliably recorded in baseline and drug-exposed conditions for a minimum of two hours. The number of active channels per larva ranged from 15 to 59 channels per experiment (median: 34, total number of experiments: 22). From most channels (90%, 774 out of 860 channels) we recorded units with bursting spontaneous activity under baseline condition (Fig 2a) and a smaller proportion (10%, 86 out of 860 channels) were units, which fired continuously. After adding KCl or PTZ to the recording chamber bath, the majority of units (in all animals) responded by increasing firing and bursting. The raw data trace in Fig 2b shows a typical seizure starting at about 10 minutes after PTZ application with a prolonged action potential burst followed by short paroxysmal action potential bursts (Fig 2c) representing a seizure pattern. An example of an EEG-recording matching a multi-unit recording during PTZ-addition is shown in Fig 3.

**Fig 4:**
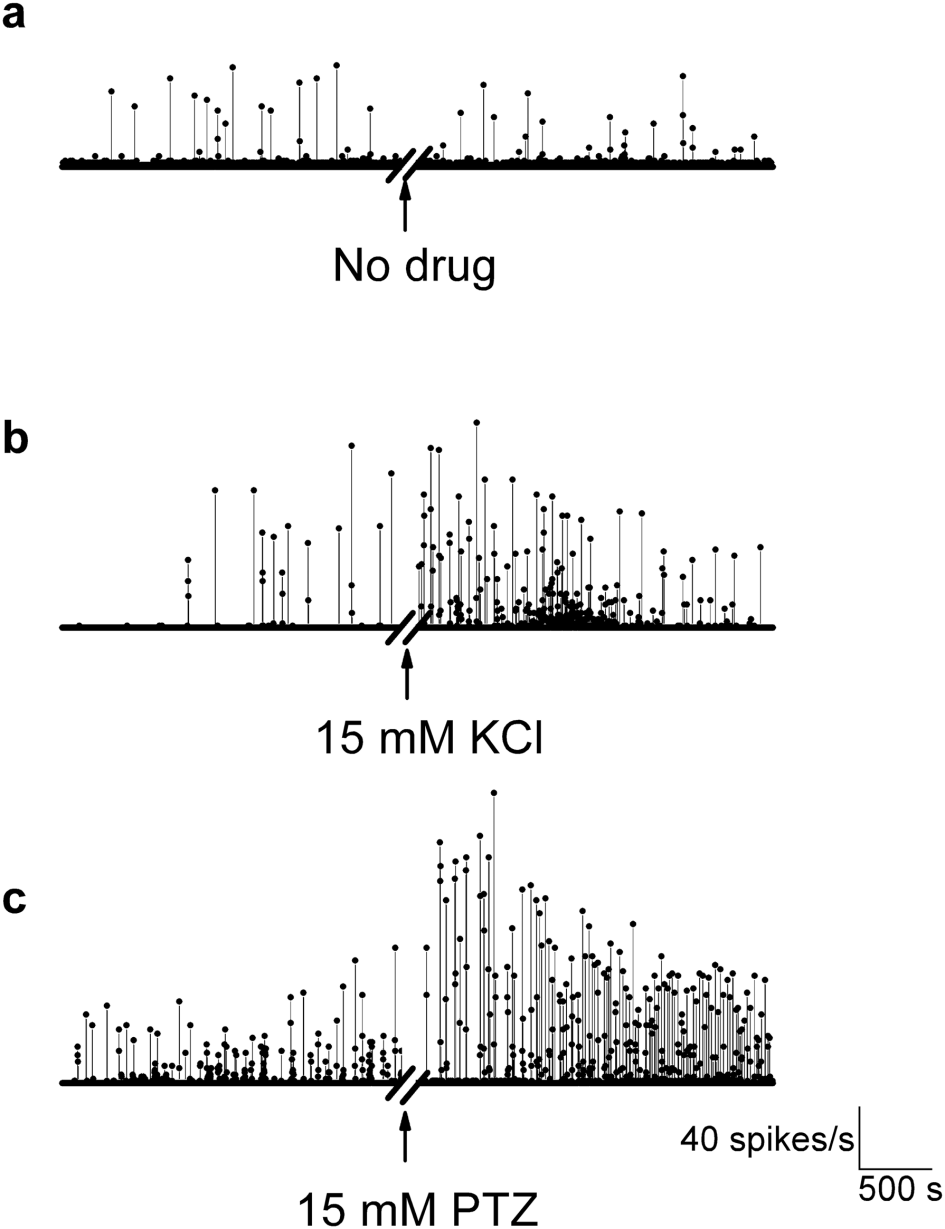
Spike rate as a function of time. Spike rate is plotted as a function of time (bin size: 1s)–one channel per condition is shown. No significant change in burst or firing rate follows a sham convulsant application (**a**). Epileptic activity is detected as increased action potential firing rate and bursting after the addition of 15 mM KCl (**b**) or 15 mM PTZ (**c**). Spike rate increases with drug addition.

To illustrate how the extracellular field activity pattern changes over time with drug addition, the spike rate was plotted as a function of time. Under control or sham condition (when no drug is added after the first 30 min of recordings), there is only steady relatively low firing and bursting (Fig 4a). However, after adding the convulsants KCl or PTZ, there is a strong increase in firing rate and burst rate (Fig 4b and 4c). This across-channel coherence in spike firing over time occurred in all experiments at all active channels.

### Recording stability

**Fig 5:**
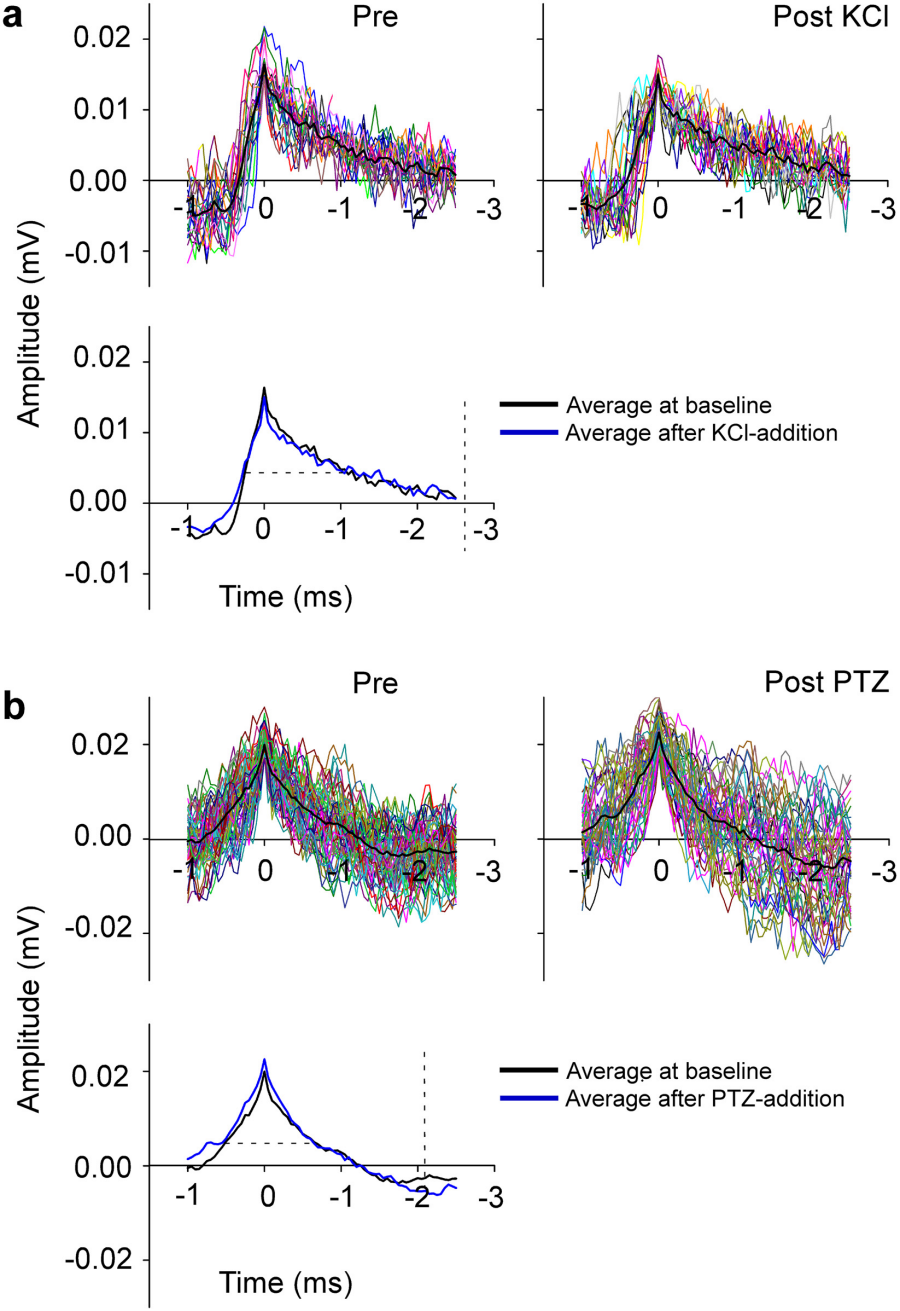
Confirmation of cell stability. Top panels in (**a)** show representative single cell recordings before and after KCl addition, top panels in (**b**) before and after PTZ addition. Bottom panels show averages of baseline and KCl recordings (**a**) and of baseline and PTZ recordings (**b**). Dashed lines indicate the width of the averaged action potential at half maximum of the amplitude and they approximate 1.3 ms in both recordings. Action potentials are stable over time as indicated by the unchanged shape of the traces.

To test recording stability between the beginning and the end of an experiment, the shape of the recorded signals was analyzed. In Fig 5, signal quality (shape and amplitude of signal) did not change between the beginning (baseline) and end of an experiment (after drug-addition). This was the case for KCl (Fig 5a) as well as PTZ (Fig 5b).

### Firing characteristics

**Fig 6:**
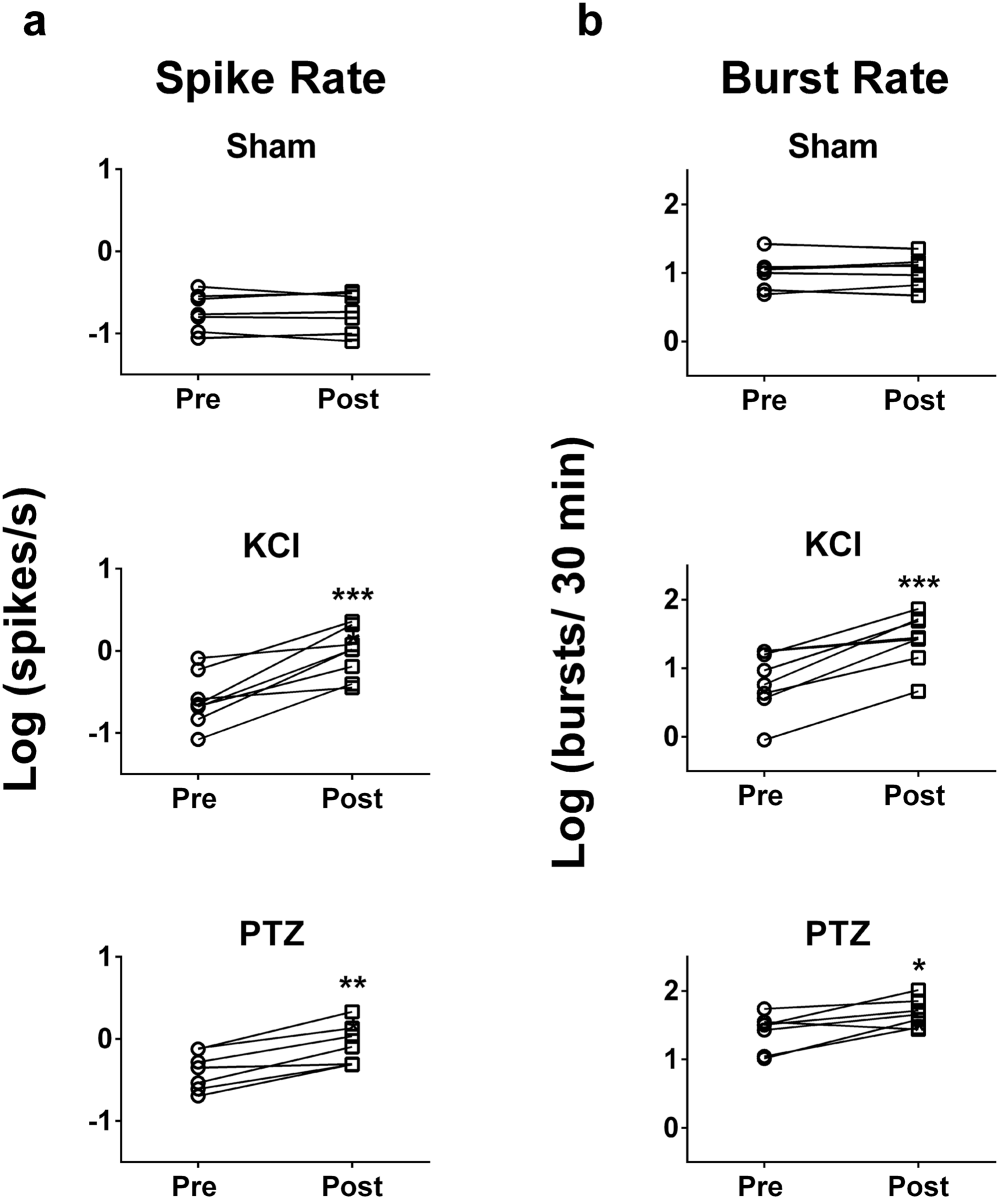
Spike rate and burst rate increase after drug-addition. **a:** Summary statistics of recordings from all active channels show a significant increase (** p < 0.01) in spike rate across all experiments following KCl (p = 0.001) or PTZ addition (p = 0.001) to the bath in contrast to the sham condition (p = 0.87). **b:** Summary statistics of recordings from all active channels show a significant increase (* p < 0.05, *** p < 0.001) of bursting across all experiments following KCl (p = 0.0006) and PTZ addition (p = 0.02) to the bath in contrast to the sham-condition (p = 0.53). The addition of the chemical convulsants KCl and PTZ leads to an increase in firing and bursting of action potentials.

In 100% of experiments (assessed per fish), the spike and burst rate increased significantly from baseline in the KCl or PTZ condition. Summary statistics including all active channels from all animals reveal that action potential firing rate increased significantly after the addition of 15 mM KCl or 15 mM PTZ to the bath (Fig 6a, sham: p = 0.87, n = 7; KCl: p = 0.001, n = 8; PTZ: p = 0.001, n = 7). Burst rate also increased significantly when both drugs were added, however it did not change in the control/ sham-condition (Fig 6b, sham: p = 0.53, n = 7; KCl: p = 0.0006, n = 8; PTZ: p = 0.02, n = 7).

### Active firing fraction and spatial coherence

**Fig 7:**
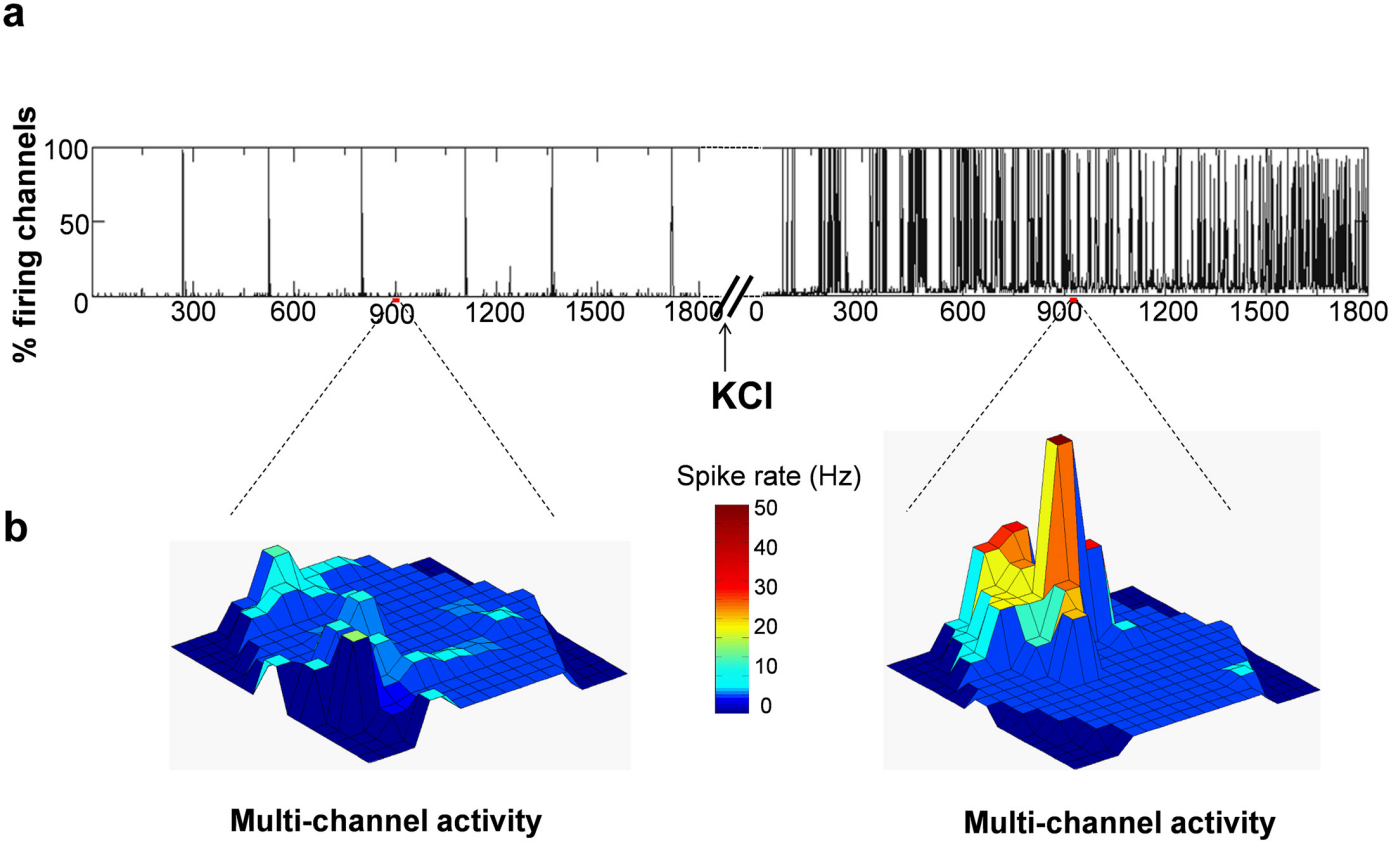
Active electrode firing fraction and spatial coherence of electrical activity. **a:** Representative firing fraction, a measure of percent firing channels per time, shows a significant increase in the number of channels that fire simultaneously after addition of KCl. **b:** The multi-channel activity is a representation of 1 sec frames displaying asynchronous firing between channels at baseline which is synchronized and highly coherent across multiple channels in the recording grid after adding KCl to the bath.

We investigated whether convulsant administration also increases firing synchrony. Fig 7a shows the active firing fraction (percent of channels firing per time) in a representative recording wherein highly repetitive synchronous firing in multiple channels is observed after KCl addition, relative to baseline. The 3D frame from the recording further illustrates how multiple channels activate simultaneously in a coherent manner after KCl, as opposed to asynchronous firing at baseline (Fig 7b).

**Fig 8:**
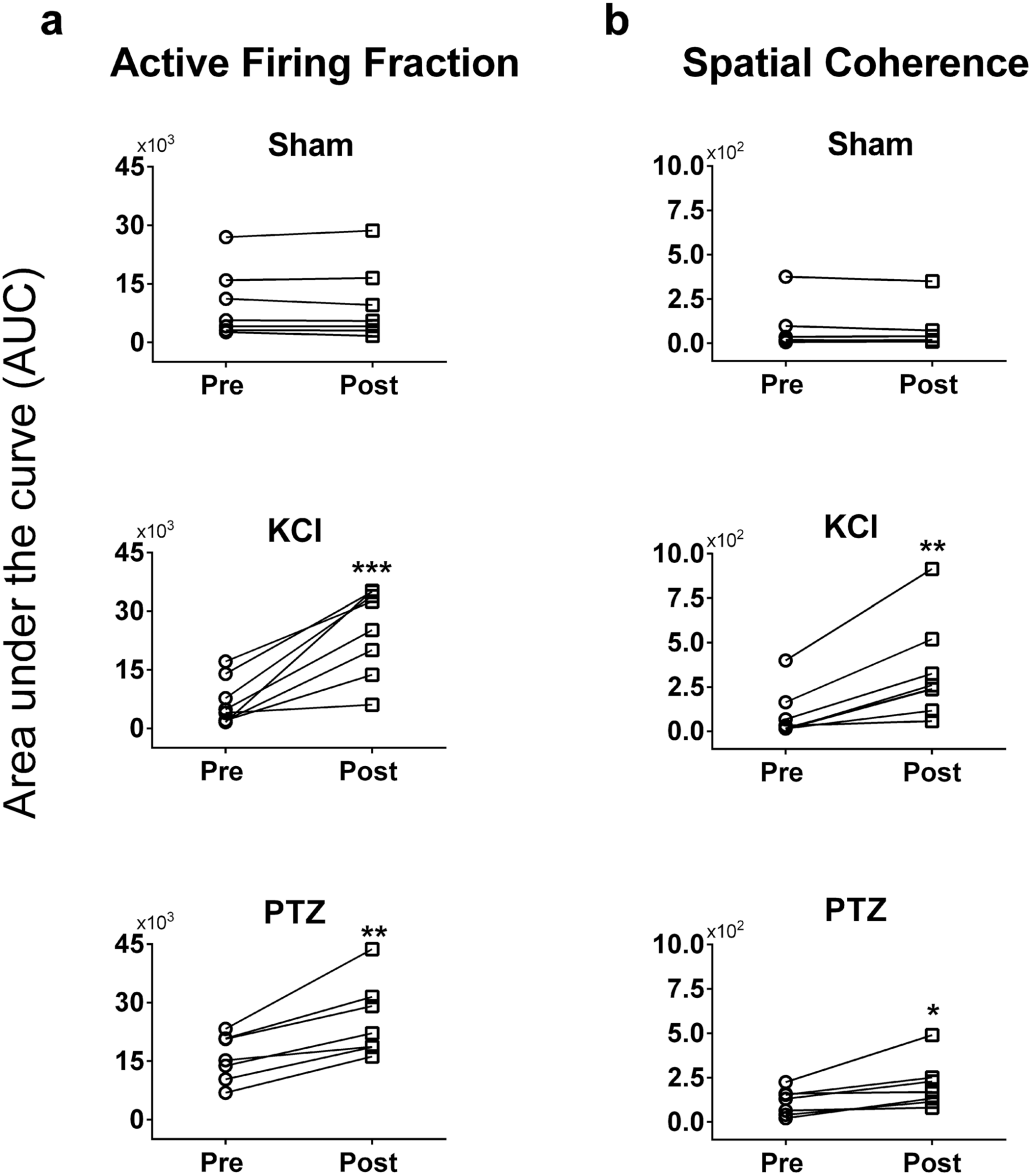
Increase in active electrode firing fraction and spatial coherence of electrical activity after drug addition. **a:** Area under the curve (AUC), a numerical integration, of the total firing fraction between baseline and post-drug periods shows significant increase (** p < 0.01, *** p < 0.001) after KCl (p = 0.0009) and PTZ (p = 0.002) addition relative to baseline. Note the similarity in the firing fraction across time when no drug (sham) was added to the bath (p = 0.86). **b:** Area under the curve of spatial coherence, a measure derived by comparing the correlation of coefficient of a central pixel with respect to its nearest-matching neighbor along time, also did not differ between baseline and follow-up in the sham condition (p = 0.19). Notably, more channels fired simultaneously indicating a significant increase (* p < 0.05, ** p < 0.01) in spatial coherence after KCl (p = 0.002) and PTZ (p = 0.02) administration.

The cumulative area under the curve (AUC) of firing fraction in the control condition did not vary significantly between the baseline and follow up (Fig 8a, p = 0.86). However, the active firing fraction in 1800 seconds after drug was significantly higher than that at baseline (KCL: p = 0.0009; PTZ: p = 0.002). Similarly, the AUC formed by the correlation of coefficient for spatial coherence pattern was similar during two consecutive 1800-second segments in the control (sham) condition (Fig 8b, p = 0.19). However, the spatial coherence between channels significantly increased with drug addition as compared to their baselines (KCL: p = 0.002; PTZ: p = 0.02) indicating a strong spatial synchrony between channels brought out by exposure to the drug.

### Seizure recording form paralyzed larvae

**Fig 9:**
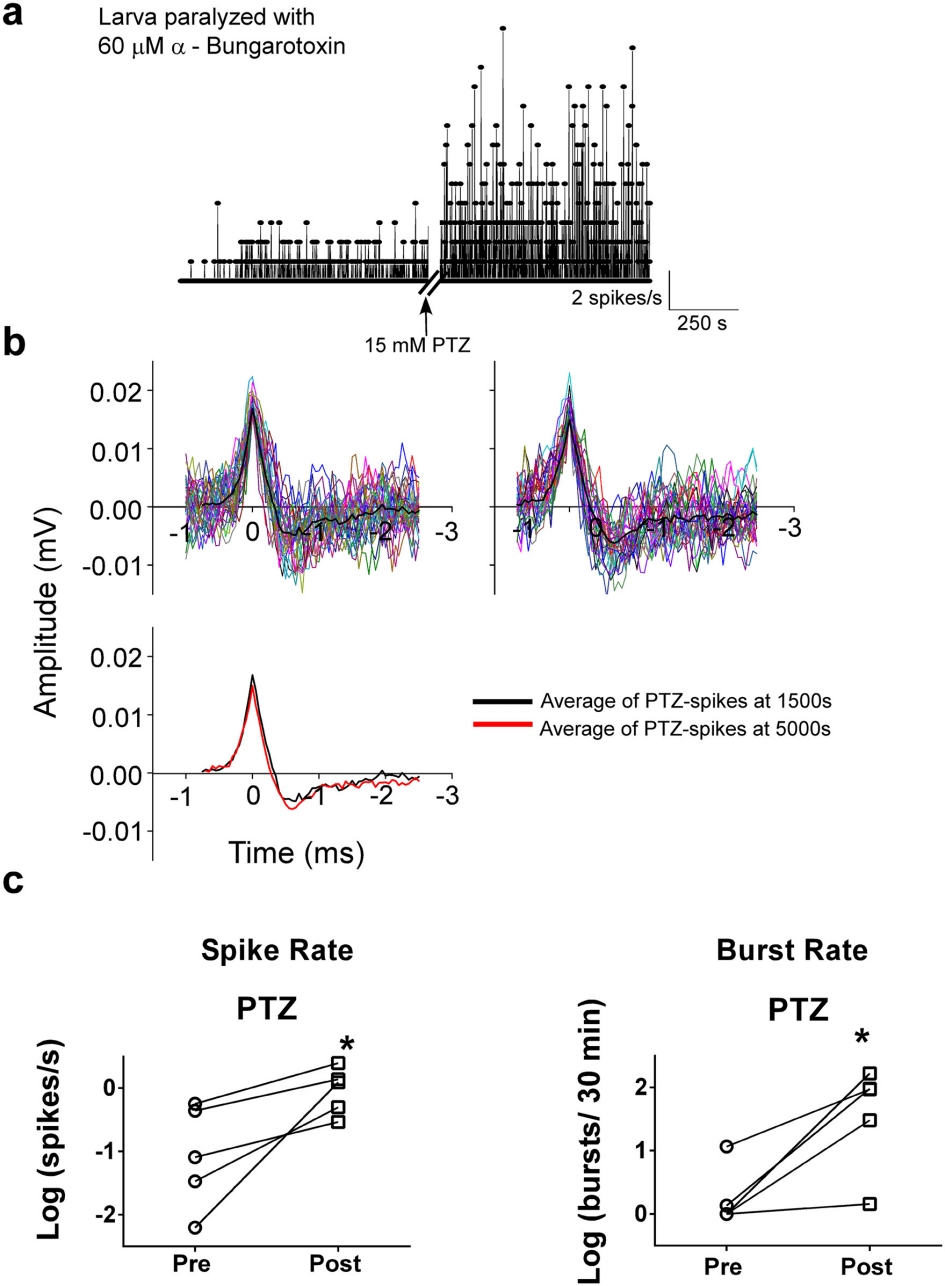
Paralyzing larvae for recordings leads to similar results described for unparalyzed animals. **a:** Spike rate plotted as a function of time (one channel shown). Recordings are made from a paralyzed larva (using 60 μM α-bungarotoxin) before and after PTZ addition. **b:** Spikes remained stable at the beginning of the PTZ addition and 3600 s later similar to recordings in larvae that were not paralyzed. **c:** Firing rate (p = 0.04, n = 5) and burst count (p = 0.02, n = 5) of recordings shown in (**a)** increase significantly (* p < 0.05) with PTZ addition in paralyzed larvae just as in unparalyzed animals.

**Fig 10:**
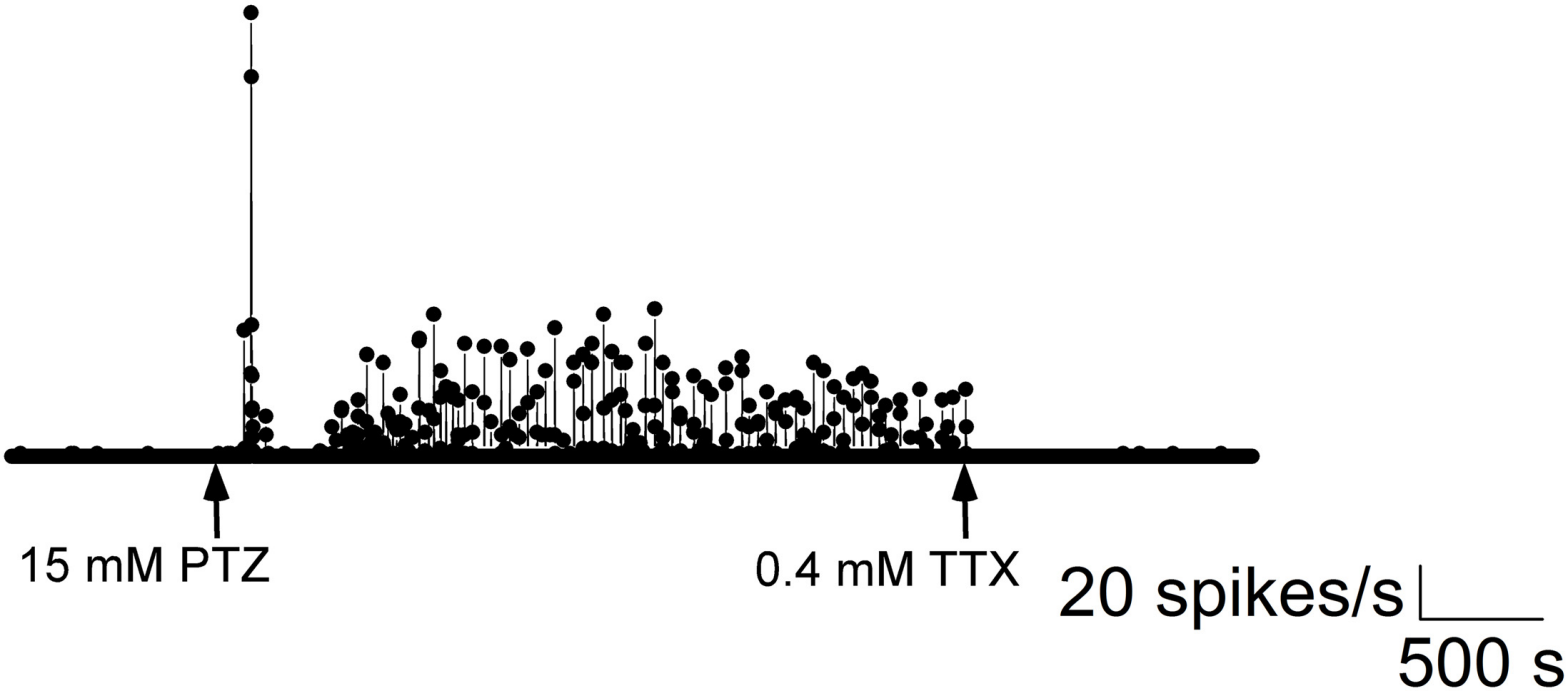
Addition of TTX to seizing larvae abolishes spiking. Spike rate plotted as a function of time (one channel shown). Recordings are made before and after PTZ addition. The addition of 0.4 mM TTX at 3900 s abolished firing. These experiments demonstrate a high likelihood that recorded signals arise from neurons firing action potentials (in contrast to myogenic potentials arising from tail-movements).

Recordings from larvae paralyzed with α-bungarotoxin showed continuous robust spiking and bursting when exposed to 15 mM PTZ similar to recordings from larvae that were not paralyzed (Fig 9a). Field potentials resembled multi-unit or single-unit action potentials after high pass filtering and remained stable from the beginning to the end of the recording (Fig 9b). Data from five fish showed significant increases in spike rate and burst rate (Fig 9c) after PTZ addition (spike rate: p = 0.04; burst rate: p = 0.02, n = 5). The addition of 0.6 mM TTX abolished firing (Fig 10), which suggests that voltage-gated sodium channels typically involved in action potential generation were blocked.

### Animal survival

All larvae survived the experiment, and were swimming freely for at least 2 hours after the experiment. Fourteen larvae were monitored for long term survival following a minimum of a two-hour recording session unexposed to either KCl or PTZ; minimum observation time after recording was 24 hours. Of these, 12 were alive at least for 24 hours (maximum 10 days), though two larvae expired between the 2-hour and 24-hour observation. All surviving larvae maintained an upright position and responded to mechanical tapping near their bodies with a startle response or swam freely (just as unexposed and unrecorded larvae) 24 hours after recording time.

## Discussion

This is the first report of multi-electrode extracranial recordings of epileptic seizures in zebrafish larvae, which allows the survival of the animal for ongoing studies. A major advantage of this recording method in larval zebrafish is the use of an array of surface electrodes to record simultaneously from multiple brain regions. As we record from multiple channels, our MEA-based method enables not only measures of changes in neuronal firing rate and pattern, but also seizure-related measures of neuronal synchrony across regions—all findings indicative of epileptic seizures [20] and provoked by common chemoconvulsants used in our experiments. In our approach, we quantify synchrony and excess electrical activity by defined parameters, such as spike rate, burst rate, and spatial coherence, in contrast to qualitative assessments of recording traces used in prior studies [1, 21–23]. In addition we note that the MEA-based recording protocol enables seizure detection by either extracellular multi-unit or EEG recordings from the same preparation.

Although this is not the only method which does not require the placement of an electrode into the larval head [6], our noninvasive protocol also minimizes potential brain injury that may provoke abnormal neuronal behavior. Another advantage of this method where the larva are restrained mechanically, is that it does not require paralytics or embedding the larvae in agar, thus further increasing the chance of survival.

MEA systems (used for electrophysiological recordings from isolated brain slices or cultured neurons) are increasingly commonly used recording systems in neuroscience laboratories and can thus be readily applied to recordings from zebrafish larvae. They are relatively straightforward to establish, since they require less noise-optimization due to mechanical stability and integrated equipment. For example, common components typically used in extracellular recording rigs, such as manipulators, head stages, and expensive isolation-vibration tables, that would each normally have to be stabilized and grounded, are not required elements of MEA recording systems. Thus, we are hopeful that our method will be adopted by investigators who currently have access to MEA equipment.

Stability during recordings from the brain was achieved as demonstrated by unchanged signal quality (no loss in amplitude or change in shape during recordings of at least one hour, see Fig 5). The skull of a larva at 4 to 5 dpf is thin enough (less than 50 μm, [24]) to record action potentials including both multi-unit and single-units. Representative examples of single units are shown in Figs 5a and 5b and 8b and the morphology and duration at half maximum of the average action potentials are falling well within the range of extracellularly-recorded vertebrate action potentials in terms spike amplitude and duration [1, 25]. The difference in shape between the action potentials shown in Figs 5 and 9b can mostly be attributed to the relative distance of the spike to the soma of the neuron, as has been reported by others [5]. Further, as we are recording from the surface of an intact fish, to address potential concerns that we might be recording myogenic signal, we applied α-bungarotoxin and showed that our results are consistent with or without paralysis of the larvae. Given the similarity in recordings between the paralyzed and non-paralyzed conditions, we conclude that our method detects neuronal signal that is not contaminated by muscle artifact.

As had been done for the previously established single-channel invasive recording method for larval zebrafish, we demonstrated that seizures are reliably provoked by the chemoconvulsants PTZ and KCl exposure. Neuronal firing and bursting increased significantly, as did spatial and temporal coherence—all parameters together (quantified for the first time in a zebrafish preparation) indicating the increased neuronal synchrony and excitability that characterizes epilepsy and correlating with the observation of behavioral seizures in larvae exposed to the same concentrations of PTZ and KCl.

### Study limitations

There are a few limitations of our method that should be noted. The electrode grid is large enough to overlap with structures of the head (e.g., eyes), or parts of the brain, such as the medulla or cerebellum that are not known to show epileptic activity in fish in contrast to the telencephalon or the tectum [1]. However, we deliberately included all brain areas for analysis, assuming that it is possible to induce epilepsy also in areas besides the telencephalon and tectum using chemoconvulsants. In future studies, one could easily exclude channels located more caudally or at the location of the eyes from data analysis, if desired.

Furthermore, we are not able to modify the electrode position to deeper areas of the brain or to very confined areas or specific nuclei within the brain. Yet, the advantage of this method is that it is non-invasive, and we prioritized this feature over recording directly of deeper structures, analogous to what is done routinely in human electroencephalography.

Electrodes in the array that we used are of low impedance, thus we are able to record EEGs, as well as action potentials under very low noise-condition. However, it is not possible to always isolate single units based on amplitude in those recordings. If desired, though beyond the scope of this, it is possible to isolate units through spike sorting based on spike shape differences.

### Future directions

As an application of this MEA recording protocol for larval zebrafish, we propose to use this method to screen for epileptic seizures, or abnormal electrical activity suggestive of a tendency to seizures, in zebrafish larval models of known and candidate epilepsy genes. In parallel with behavioral seizure observations, we will use this method to confirm the presence of electrophysiological abnormalities in such models and their response to candidate antiepileptic drugs. Since our method is noninvasive and many larvae survived experiments at least for two days (maximum ten days so far), we have the possibility as well to conduct longitudinal experiments, to either record serially form the same fish, or to enable the larva to mature after recording and observe long-term effects of seizures or other manipulations conducted during the MEA recording session.

In conclusion, we have established a noninvasive multi-electrode recording technique that can be adopted by many neuroscience laboratories to detect seizures in larval zebrafish, plausibly coupled with high-throughput antiepileptic drug screening protocols, as has been proposed in analogous zebrafish epilepsy model research [4, 10, 18]. Our noninvasive recording protocol affords the ability to conduct serial experiments on the same larvae. Coupled with genetic techniques and behavioral analysis of genetic models of epilepsy, this method will be useful in the establishing and evaluating genetic models in which to test novel antiepileptic drugs for genetic and other forms of epilepsy.
