# Correction: Microarray Noninvasive Neuronal Seizure Recordings from Intact Larval Zebrafish

**DOI:** 10.1371/journal.pone.0159472

**Published:** 2016-07-14

**Authors:** Michaela Meyer, Sameer C. Dhamne, Christopher M. LaCoursiere, Dimira Tambunan, Annapurna Poduri, Alexander Rotenberg

There is an error in Fig 2 on the scale bar: the correct value on the y-axis should be 0.05 mV instead of 50 mV. There is an error in Fig 3 on the scale bar: the correct value on the x-axis should be 0.5s instead of 500s. Please see the corrected Figs 2 and 3 here.

**Fig 2:**
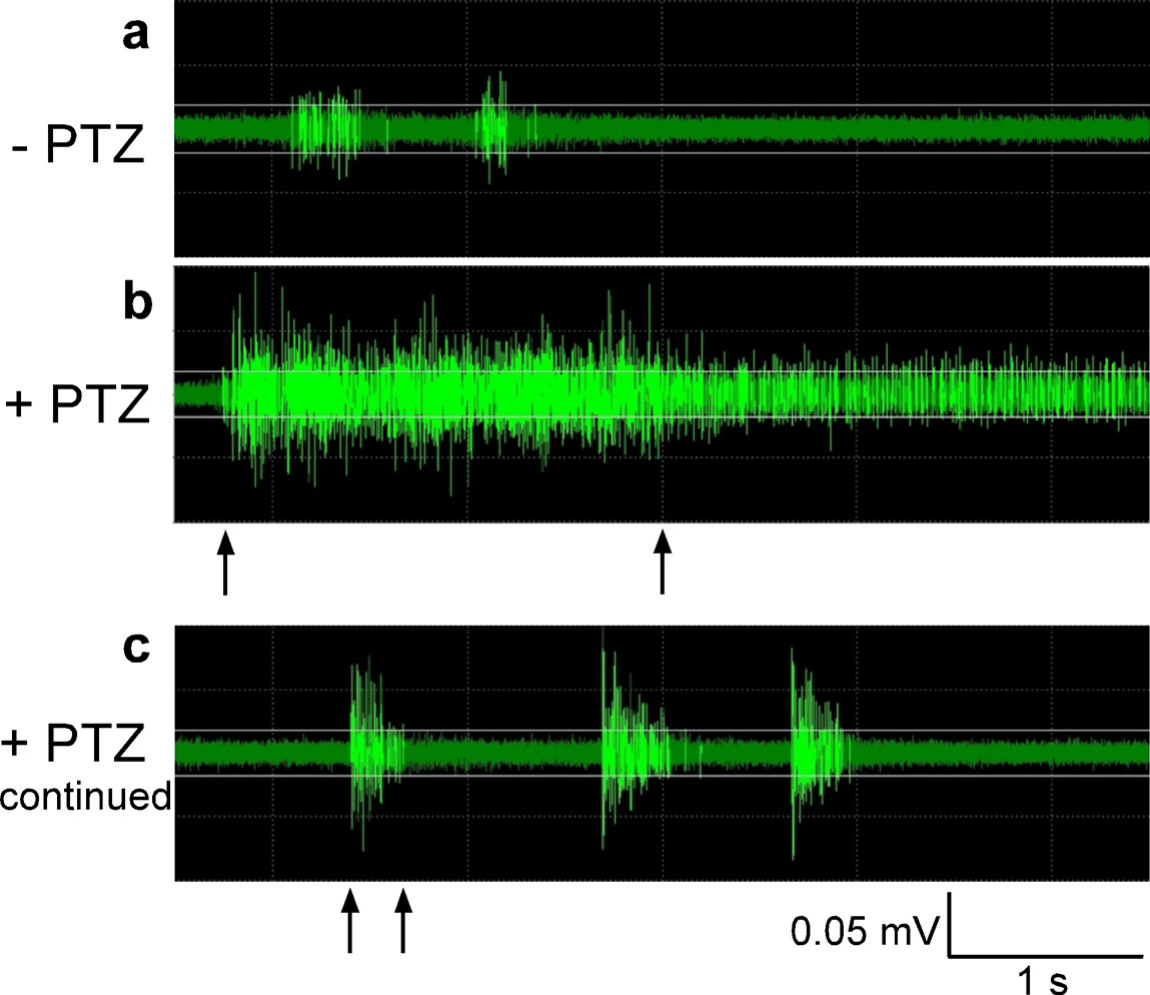
Provoked seizures in 5-second raw data traces of action potentials. a: Baseline activity before PTZ addition. b: A typical seizure starting with a prolonged action potential burst 1–2 minutes after PTZ application. c: Seizures continue as short paroxysmal action potential bursts. PTZ addition leads to a seizure firing pattern. Markings denote sustained (> 2 s; b) versus short (< 500 ms; c) bursting.

**Fig 3:**
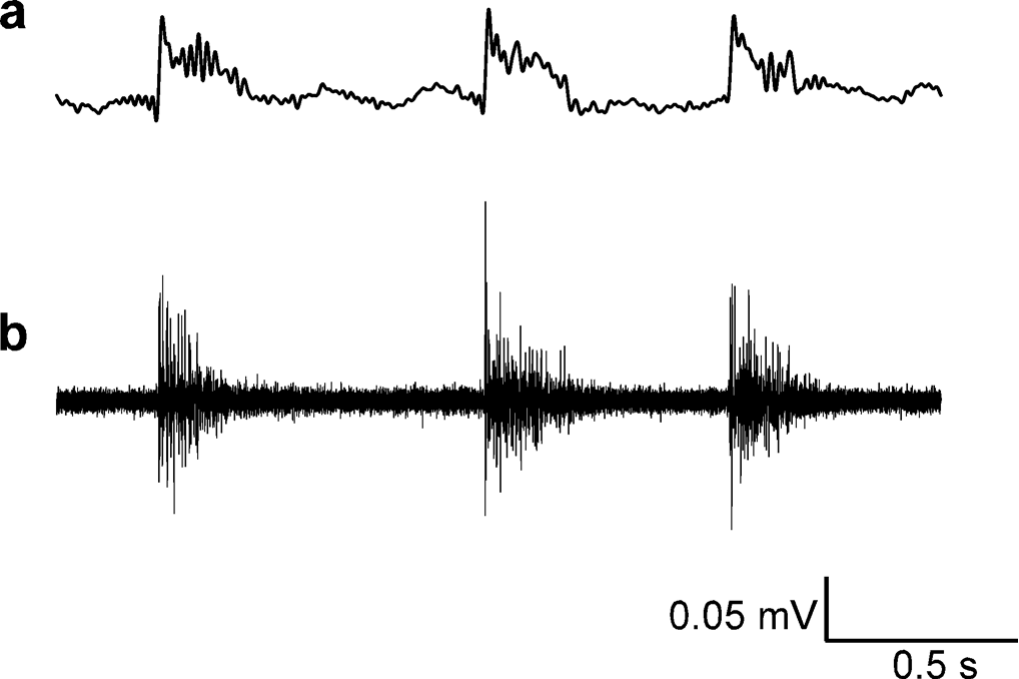
EEG and action potential recordings. Both EEG and action potential recordings from zebrafish larvae can be obtained simultaneously and correlated with each other. These recordings are taken from one channel following PTZ administration. a: Data are filtered to show an EEG (bandpass filter 1–25 Hz). b: Corresponding multi-unit activity (high pass filter at 100 Hz).
